# Editorial: A new era: shaping women's metabolic health, fertility, and sex-related cancers

**DOI:** 10.3389/fnut.2024.1371975

**Published:** 2024-03-13

**Authors:** Costanza Maria Cristiani, Gustavo Cernera, Marzia Di Donato

**Affiliations:** ^1^Neuroscience Research Center, Department of Medical and Surgical Sciences, University “Magna Graecia,” Catanzaro, Italy; ^2^Dipartimento di Medicina Molecolare e Biotecnologie Mediche, Università di Napoli Federico II, Naples, Italy; ^3^CEINGE-Biotecnologie Avanzate, Naples, Italy; ^4^Dipartimento di Medicina di Precisione, Università della Campania “L.Vanvitelli“, Naples, Italy

**Keywords:** fertility, diet, breast cancer, ovarian health, obesity

## 1 Introduction

Metabolic health, resulting from genetic, constitutional, and environmental factors, differs between the two sexes. Similarly, metabolic disorders and their associated risk factors differently affect men and women, either being peculiar only for women or showing sex-related differences in incidence and outcome. Therefore, the identification of altered pathways in female subjects, as well as the design of specific therapeutic strategies, is mandatory for the development of precision medicine.

In this Research Topic, several aspects of female health issues are discussed, with particular focus on the impact of nutrition on female diseases and cancers.

## 2 Effects of nutrition on female fertility and related issues

Although there is evidence showing that genetic aberrations can affect women reproductive capabilities (1, 2), nutritional habits could be an important and pivotal modifiable factor impacting female fertility. Indeed, metabolic disorders have been proposed to affect woman's fertility either by directly dampen oocytes or by indirectly interfering with hormonal feedbacks (3). Moreover, an imbalanced food intake has been associated with the onset of gestational diabetes mellitus (GDM) (4).

In a case-control study, Ziaei et al. evaluate the association between ovarian reserve and diet quality, assessed by Diet Quality Index–International (DQI-I) score, reporting that women with diminished ovarian reserve (DOR) show lower DQI-I scores. Accordingly, the authors also demonstrate that a high DQI-I score decreases the odds of DOR and that a greater adherence to DQI-I is associated to a higher antral follicular count in women with DOR.

Another case-control study by Ghasemisedaghat et al., analyzing the association between fertility diet score and the odds of endometriosis in Iranian women, reports that the assumptions of multivitamins and vegetables may be protective against endometriosis. Conversely, high consumption of animal proteins, heme iron, and glycemic load could be responsible for endometriosis.

Trace elements are known to play specific roles in diabetes pathogenesis, although their role in GDM is poorly understood. Deng et al. address the question in a prospective cohort study investigating how the exposure to both essential and toxic minerals during early pregnancy affects GDM development. The authors demonstrate that serum concentrations of calcium, copper, zinc and iron are associated with increased odds of GDM. Notably, the authors also evaluate the joint effect of the mixture, reporting that zinc, copper and cadmium are the strongest contributors to GDM development.

In their systematic review and meta-analysis, Liu et al. discuss the capability of N-acetylcysteine (NAC) supplement to affect metabolic parameters in women with PCOS and evaluate whether NAC may represent an effective alternative to metformin. By analyzing 11 randomized controlled trials, the authors report that NAC is able to improve metabolic parameters in women with PCOS, such as anthropometric measures, fasting blood glucose and insulin and cholesterol levels. However, the authors demonstrate that NAC efficacy is equal or lower to metformin.

In their article, Xing et al. analyze the role of SIRT1 in embryo development, particularly in the blastocyst stage after fertilization, giving strength to the field of application related to aging and diseases related to SIRT1.The authors observe that luteolin, present in fruits and vegetables and in agri-food by-products (5), delays postovulatory oocyte aging and controls organelles distribution and function through the up-regulation of SIRT1 activities during postovulatory oocyte aging.

## 3 Association between nutrition, inflammation, breast and ovarian cancer

The link between cholesterol metabolism and inflammation is already reported in some genetic disorders (6–9). However, metabolic disorders of adipose tissue are also known to stimulate cancer development and progression by eliciting the release of several pro-tumoral factors such as inflammatory mediators, hormones and growth factors (10, 11). This is of particular interest in women, since two typically female tumors, breast and ovarian cancer, have been associated with obesity.

In their cross-sectional study, Hajmir et al. specifically address the role of ultra-processed food (UPF). Particularly, the authors demonstrate that a high intake of UPF is associated with both a high risk of displaying a metabolically unhealthy obese phenotype and higher concentrations of inflammatory markers. The impact of UPF on female health is discussed also by Shu et al.. In their systematic review and meta-analysis involving six papers and more than 460,000 patients in total, the authors find that UPF consumption increases the odds of breast cancer. Moreover, such an association shows a linear pattern, in which the risk linearly increases with UPF consumption.

On the other hand, the association between obesity and breast cancer is discussed in the meta-analysis by Chen et al.. In this Research Topic, the authors underline the connection between central obesity (specifically determined by waist circumference and waits/hip ratio) and pre- and post-menopausal breast cancer. In particular, central obesity could result in a risk for both Estrogen receptor (ER) positive and negative Breast cancer types (12, 13). Thus, nutritional interventions could reduce chronic inflammation and abnormalities in visceral fat-related metabolism, such as elevated insulin-like growth factor-1 levels and hyperinsulinemia, which can also contribute to this association.

Meta-analysis by Zhang et al., taking into account more than 40 studies, focuses on the relation between fat intake and ovarian cancer, the most lethal gynecological malignancy, given the lack of early symptoms and specific biomarkers. Particularly, the authors observe that a higher daily intake of total fat, saturated fat, animal fat, and cholesterol and higher levels of serum triglycerides are significantly associated with an increased risk of ovarian cancer.

## 4 Conclusion

Nutrition has been shown to exert a pivotal role in determining reproductive health in women. As an example, insulin resistance promoted by excessive caloric intake induces androgen over-synthesis and generates an unfavorable biochemical environment within ovaries, causing oligo-anovulation and hyperandrogenism typically observed in PCOS (14). Therefore, studies assessing the effects of nutrition on female fertility might provide useful support to current infertility treatments. Similarly, by eliciting hormone release, an unbalanced diet may promote cancer development, particularly hormone-sensitive tumors such as ovarian and breast cancer. Given the high incidence of these cancers and the related mortality, particularly when they progress toward malignant stages of the disease, studies aimed at preventing cancer through nutritional interventions are of particular interest.

## Author contributions

CMC: Writing – original draft, Writing – review & editing. GC: Writing – original draft, Writing – review & editing. MD: Writing – original draft, Writing – review & editing.
